# Depressive Symptom and Associated Factors Among School Adolescents of Urban, Semi-Urban and Rural Areas in Bangladesh: A Scenario Prior to COVID-19

**DOI:** 10.3389/fpsyt.2021.708909

**Published:** 2021-09-28

**Authors:** Afifa Anjum, Sahadat Hossain, M. Tasdik Hasan, Sayma Islam Alin, Md. Elias Uddin, Md. Tajuddin Sikder

**Affiliations:** ^1^Department of Public Health and Informatics, Jahangirnagar University, Dhaka, Bangladesh; ^2^Jeeon Bangladesh Ltd., Dhaka, Bangladesh; ^3^Public Health Foundation, Dhaka, Bangladesh; ^4^Department of English, University of Dhaka, Dhaka, Bangladesh

**Keywords:** depressive symptom, school adolescents, socio-demography, lifestyle, Bangladesh

## Abstract

**Background:** The purpose of this study was to investigate the prevalence of depressive symptom and the factors associated with this condition among urban, semi-urban and rural adolescents in the Dhaka district of Bangladesh.

**Methods:** A cross sectional study using two stage cluster sampling procedure was performed. A self-administered questionnaire was conveyed to 2,355 adolescents from nine secondary schools of Dhaka district of Bangladesh. Of the respondents, 2,313 completed the nine item Patient Health Questionnaire (PHQ-9). Besides, sociodemographic information, self-reported body image as well as modification of Leisure Time Exercise Questionnaire (LTEQ) and WHO Global PA Questionnaire (GPAQ) were used to determine the sociodemographic and lifestyle factors associated with depressive symptom among adolescents.

**Results:** A total of 30.1% adolescents were found to be suffering from moderate to severely severe depressive symptom. Females (60.8%) were found suffering significantly more than males (39.2%). Sociodemographic factors, for example, residential setting and family size were found significantly associated with depressive symptom among adolescents. Adjusted estimate of logistic regression shows that physical inactivity (AOR: 1.44; 95% CI: 1.14–1.84), >2 h/day screen time (AOR: 1.68; 95% CI: 1.39–2.03), sleep dissatisfaction (AOR: 3.23; 95% CI: 2.64–3.96), and underweight body image perception (AOR: 2.30; 95% CI: 1.70–3.13) were significantly associated with depressive symptom among adolescents.

**Conclusions:** Among urban, semi-urban and rural school adolescents in Dhaka, Bangladesh, depressive symptom is quite prevalent. To lessen the spread of depressive symptom among Bangladeshi adolescents, urgent steps should therefore be taken.

## Introduction

Adolescence is a period of life preceded by childhood and followed by adulthood and is usually considered to be between the ages of 10 and 19. During this time an individual undergoes major physical, mental, and behavioral changes (1) and the individual's lifestyle habits and health behaviors are developed (2). In addition, in this phase of life special health and developmental needs arise. Adolescence can be a creative stage of life for some individuals as well as a catastrophic time for some others (3). For many teens, the increasingly digitized and socially competitive environment is making the world more and more complex (4). However, the special needs of the adolescents are often ignored, leading to the exacerbation of the complexities of their social and family lives (5). Adolescents around the globe are in constant risks of developing multiple forms of health issues. Among all adolescent diseases, the ones related to their mental health are actually of great interest to the entire world and as such have received special attention from all stakeholders (6). Studies have shown that at least 20% of individuals encountered a common mental illness sometime between the ages of 12–24, and a higher prevalence was observed in the 18–24 age group than in the 10–17 age group (7, 8).

With more than 264 million people affected, depressive disorder is a widespread public health concern worldwide (9). In spite of some methodological variations in epidemiological research, 4.4% of the world's adult population are reported to suffer from depressive disorders (10). It can become a serious health condition, especially when it is long-lasting and of moderate to extreme severity. Depressive disorder can cause great distress to and poor functioning of the affected individual at work, at school and in the family. It can, at its worst, lead to suicide, which accounts for nearly 800,000 deaths per year and is the second leading cause of death among people aged 15–29 (9).

Depressive disorder is a significant adolescent psychiatric illness that can also affect an adolescent's social functioning, family relationships and academic performance (11). These issues may become recurrent, leading to behavioral and drug use disorders that cause ~40.5% of adolescent disability adjusted life years (DALYs) (12). Around 20% of teenagers experience a mental health problem, most frequently depression (13). In a systematic review of Silva et al. it was found that in Japan, children and adolescents have greater depressive tendencies, and this disorder may increase in many countries every year (14). In spite of its extreme effects, teenage depression typically remains under- reported, under-diagnosed and under-treated (15). A dynamic combination of social, psychological and biological influences results in depressive disorders. People who have endured negative life experiences are more likely to develop depression (9). In addition to gender and genetics, low parental warmth, high levels of maternal aggression and escalating adolescent-parent conflict are significant factors correlated with depression in adolescents; however, perceived alienation by peers, parents, and teachers predicts a rise in depressive symptom in children and adolescents (16).

About thirty six million adolescents live in Bangladesh, making up 22% of the population (17). However, only a few studies have been conducted to investigate depressive symptom among Bangladeshi adolescents (18–20). These studies are not enough to demonstrate the burden of depressive symptom among adolescents in the country. Moreover, these studies were conducted upon either urban or urban slum and rural or urban and semi-urban settings. None of the studies to date has addressed depressive symptom of adolescents in the urban, semi-urban and rural settings of Bangladesh. Given the immense gravity of the issue, this study has been conducted among urban, semi-urban and rural school adolescents in Dhaka, Bangladesh in order to find out the prevalence of depressive symptom among school adolescents and the factors associated with such symptoms.

## Methods

### Study Design and Setting

**Figure 1:**
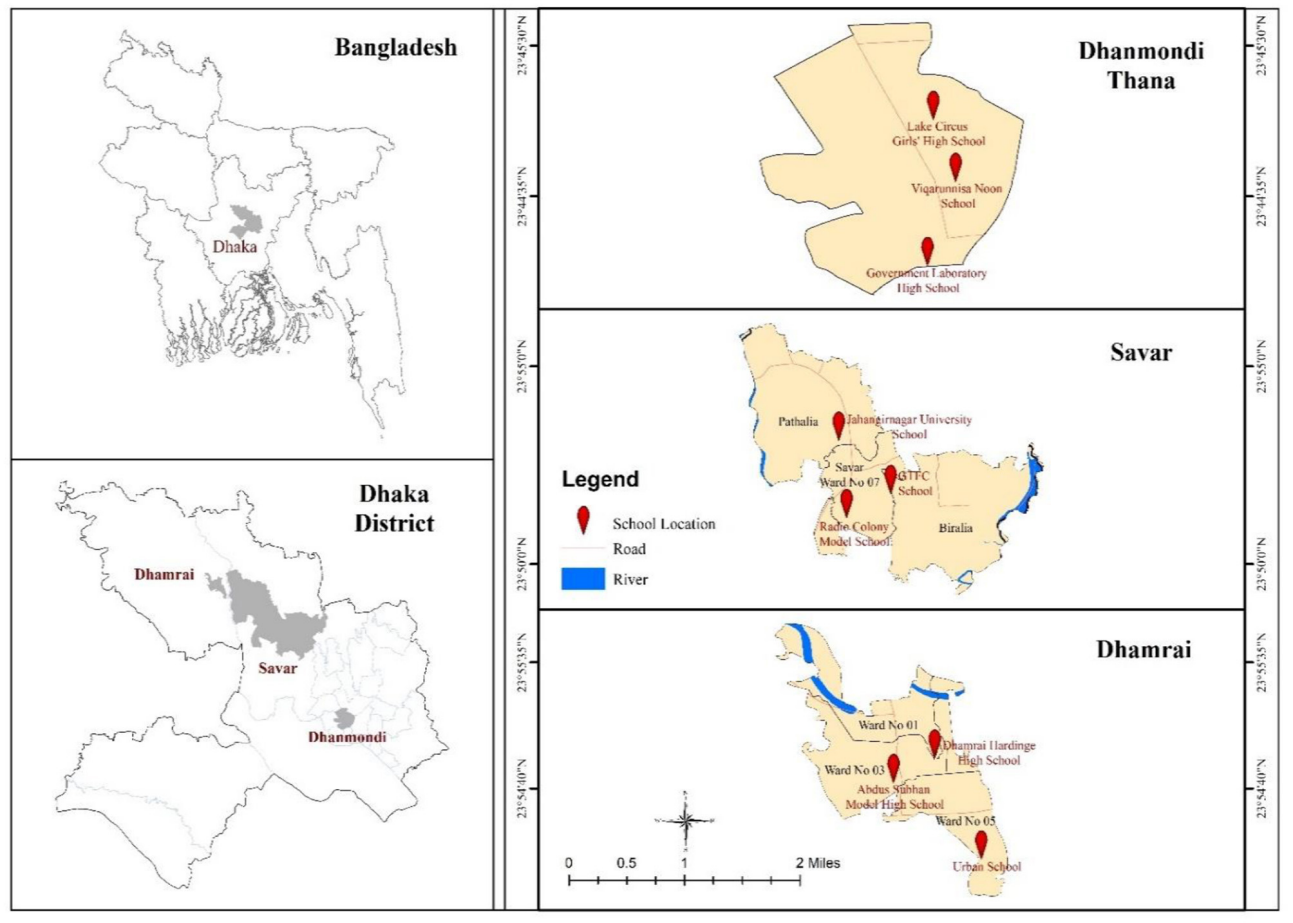
Location map of the study area.

This cross-sectional study was conducted between January 2019, and February 2020 in three different areas—urban, semi-urban, and rural (Figure 1). In this study, Dhanmondi thana in Dhaka city was the urban area, Savar thana, 24 kilometers northwest of Dhaka, was the semi-urban area, and Dhamrai Upazila which is about 40 kilometers north west of the capital Dhaka was the rural area. These three areas were purposefully chosen for this study, taking into account the intra-thana homogeneity of the population.

### Study Procedures and Sample

Initially all the secondary schools of the urban, semi-urban and rural areas were listed and it was found that the number of secondary schools in Dhanmondi, Savar and Dhamrai were 16, 14, and 16 respectively. Three schools were purposefully chosen from each region, taking into account the diverse socio-economic context of the participants and their accessibility to the research team. The study population in this study consisted of adolescents of grades 8–10 (12–17 y), since grades 6–10 are considered secondary education at schools in Bangladesh. Following a two-stage cluster sampling technique with a 10% non-response rate, a sample of 2,355 was calculated for this study. Two stage cluster sampling technique was used in this study since the study population were from diverse clusters of different socioeconomic backgrounds, and collecting data from all units inside the clusters was not only expensive but also time-consuming. Prior to field data collection, the research team collected a student list of all the enrolled students of grades 8, 9, and 10 from the respective school authorities. After that, with the approval of the headmasters/principals of the schools as well as the teachers of respective classes, the research team went to classrooms and explained the rationale and aim of the study to the students. Students were also told that they would need to seek permission from their parents for taking part in the study. With informed consent of their own as well as of their parents, 2,313 students completed the survey questionnaire (98% response rate) in the classroom under the direction of the lead researcher. A teacher and a member of the study team were both present in the classroom to track the progress of the survey and to answer any questions or to resolve any complaints of the participants. Students who did not consent to take part in the study engaged in activities of their choice during that time. The respondents of this study can be considered representative of the whole adolescent population in the urban, semi-urban and rural areas in Bangladesh, since the urban, semi-urban and rural settings of the country share the same demographic and socio-economic conditions.

### Instruments and Outcome Measures

To measure the level of depressive symptom of the participants, this study used a validated Bangla version of the nine-item Patient Health Questionnaire (PHQ-9) corresponding to the diagnostic criteria of symptoms for major depressive disorders as stated in the Diagnostic and Statistical Manual of Mental Disorders, Fourth Edition (21). The PHQ-9 possesses good sensitivity (88%) and specificity (94%) for measuring the severity of depression among both the clinical and general population samples (22). Findings of as many as at least 26 studies show that the PHQ-9 also had high sensitivity (89.5%) and good specificity (78.8%) for detecting major depressive symptom among adolescents, and it has almost similar sensitivity and specificity for detecting depressive symptom among adults (23). Also, the sensitivity and specificity of this tool are quite similar to those of other depression screening tools tested among adolescents in primary care: Beck Depression Inventory (sensitivity 91%, specificity 91%), PHQ-9 modified for adolescents (sensitivity 73%, specificity 94%) and the Short Mood and Feelings Questionnaire (sensitivity 80%, specificity 81%) (24–26). Thus, the PHQ-9 turns out to be a promising screening tool for use among adolescents. In this study it has also been found that the PHQ-9 is a highly reliable (Cronbach's α = 0.83 [95% confidence interval (CI) of intra-class correlation coefficient 0.80–0.86]) scale for Bangladeshi adolescents.

The participants were asked how often they experienced each of the depressive symptom, with four response options: 0 = not at all, 1 = several days, 2 = more than half the days and 3 = nearly every day over the last 14 days. The response options were also treated as a continuous ordinal measure. Hence, the range of scores for the PHQ-9 was 0–27. The cut-off points for the categorization of depressive symptom were as follows: 0–4 = normal, where no depressive symptom appeared, hence no depression treatment was required; 5–9 = mild depressive symptom, where psychological support and educational counseling were required; 10–14 = moderate depressive symptom, where clinical judgement about treatment was needed based on the subject's duration of symptom; 15–19 = moderately severe depressive symptom, where a combination of treatment with drugs and therapy was needed; and ≥20 = severely severe depressive symptom, where urgent specialized treatment with drugs and therapy was required.

During data collection and analysis, the respondents who were found to be suffering from moderate to severely severe depressive symptom, were contacted through school authority by the research team for taking treatment from registered psychiatrists/ psychologists/ psychotherapists.

### Other Measures

#### Socio-Demographic Information

Data on sex, age, grade in school, birth order, parent's educational level, number of family members and residential setting were collected from the adolescents.

#### Lifestyle

Physical activity (PA), screen based sedentary behavior (SBSB) and sleeping status were considered for determining lifestyle factors of the adolescents. Three different PA levels were incorporated in the questionnaire: low-level PA (unintentional walking <30 min/d), moderate PA (walking or meditation/yoga ≥30 min/d) and vigorous PA (jogging, cycling, playing sports or gym workouts ≥60 min/d) (27). Regarding SBSB, adolescents were asked if they spent time on social media like Facebook, Twitter, and Instagram, and watched videos on YouTube or watched movies or sports. The pattern of their viewing videos/movies/sports and spending time on social media were further categorized according to hours per day. High leisure screen time was described as >2 h/d, which is in line with the commonly used screen time guideline (18).

### Statistical Analyses

Data were analyzed using descriptive statistics as well as inferential statistics. To identify any significant relationships between the study variables, test statistics such as the χ^2^ test were used. Logistic regression models were used to detect any association of study variables with the outcome variables. In regression analysis, data were adjusted for various factors and were reported for the adjusted odds ratios (AORs) with 95% CIs. The level of significance was set at *p* < 0.05. χ^2^ test and logistic regression have been conducted considering depressive symptom as the dependant variable, while socio-demographic variables (gender, age, grade, residential setting, etc.) and lifestyle variables (PA involvement, social media usage, sleep habit etc.) were considered as independent variables. The Statistical Package for the Social Sciences software for Windows, version 22.0 (IBM, Armonk, NY, USA) was used to analyse all data.

## Results

**Figure 2:**
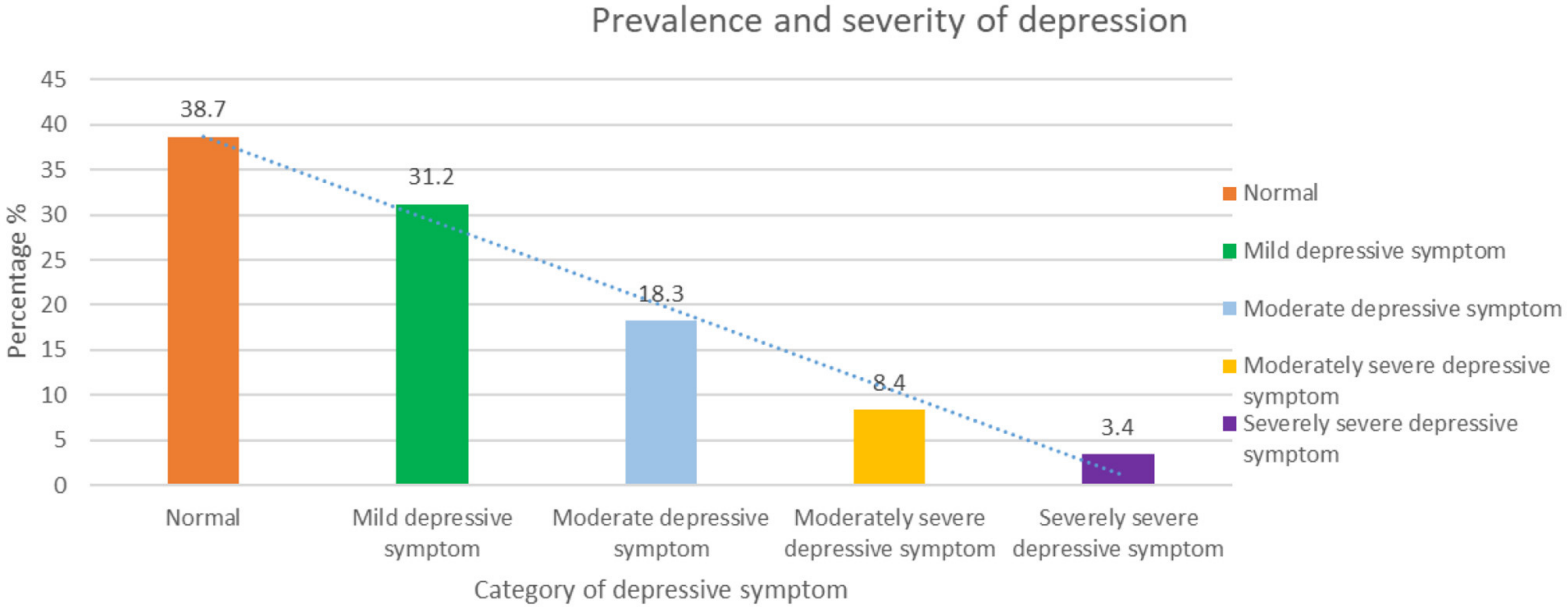
Prevalence and Severity of Depressive Symptom.

The prevalence and severity of depressive symptom among the participants are shown in Figure 2. It was found that 18.3% of the participants were suffering from moderate depressive symptom and 8.4% from moderately severe depressive symptom at the time of data collection, while only 3.4% of the participants suffered from severely severe depressive symptom, whereas 38.7% were in a normal state regarding depressive symptom. Additionally, 31.2% of the participants were found to suffer from mild depressive symptom.

### Association of Socio-Demographic Variables With Students' Depressive Symptom

Table 1 shows the association of socio-demographic variables with depressive symptom among the study participants. It demonstrates that more female school adolescents (60.8%) were suffering from depressive symptom than their male counterparts (39.2%), and this finding is statistically significant (χ^2^ = 33.72; *p* < 0.001). This study found a significant (χ^2^ = 26.03; *p* < 0.001) relationship between respondents' age and depressive symptom. The 15-year-old students (34.1%) were suffering from depressive symptom more than any other age groups, and they were followed by the age groups of 14 years (27.3%), 16 years (19.1%), and 13 years (13.2%). School adolescents of class 9 were found to have more depressive symptom (45.4%) than those of other classes, with 29.3% of the class 8 respondents and 25.3% of class 10 respondents having depressive symptom. Again, student's grade was found to be statistically significant (χ^2^ = 18.70; *p* < 0.001) with depressive symptom. Of the students who reported to have depressive symptom, their father's level of educational qualification was reported to be graduation or above by 57.7%, secondary or higher secondary by 30.3% and primary by 12%. Again, of the students who were found to have depressive symptom, their mother's level of education was reported to be secondary and higher secondary by 46.4%, graduation or above by 37.7% and primary education by 16.0%. Analysis shows that students' family size was significantly (χ^2^ = 4.30; *p* = 0.038) associated with depressive symptom. The students who had ≤4 family members were suffering more from depressive symptom (58.8%) than students having ≥5 family members (41.2%). In terms of residential area, among the total students who were found to have depressive symptom, 43.7% were from urban, 34.9% from semi-urban and 21.4% from rural areas, and this result is statistically significant (χ^2^ = 54.13; *p* < 0.001).

**Table 1:** Association of socio-demographic variables with students' depressive symptom.

**Variables**	**Depressive symptom^a^**	
	**Frequency (% within column)**	**χ^**2**^ value (*p*)**
**Gender**		
Male	273 (39.2)	33.72 (<0.001)^**^
Female	423 (60.8)	
**Age in years**		
12	22 (3.2)	26.03 (<0.001)^**^
13	92 (13.2)	
14	190 (27.3)	
15	237 (34.1)	
16	133 (19.1)	
17	22 (1.0)	
**Student grade**		
Class 8	204 (29.3)	18.70 (<0.001)^**^
Class 9	316 (45.4)	
Class 10	176 (25.3)	
**Birth order**		
1st	348 (50.0)	2.35 (0.309)
2nd	229 (32.9)	
≥ 3rd	119 (17.1)	
**Father's level of education (*****n*** **=** **1,450)**		
Primary education	55 (12.0)	4.21 (0.122)
Secondary/Higher secondary education	139 (30.3)	
Graduation/above	265 (57.7)	
**Mother's level of education (*****n*** **=** **1,486)**		
Primary education	75 (16.0)	0.86 (0.649)
Secondary/Higher secondary education	218 (46.4)	
Graduation/above	177 (37.7)	
**Total no of family members**		
≤4 members	409 (58.8)	4.30 (0.038)^*^
≥5 members	287 (41.2)	
**Residence**		
Urban	304 (43.7)	54.13 (<0.001)^**^
Semi-urban	243 (34.9)	
Rural	149 (21.4)	

a *The cut-off of PHQ-9 ≥10 is used for the depressive symptom analysis*.

* *The p value is significant at p = 0.05*.

** *The p-value is significant at p < 0.01*.

### Association of Lifestyle Variables With Students' Depressive Symptom

Table 2 demonstrates the relationship between lifestyle variables and depressive symptom. Lifestyle variables are divided into three categories, namely PA, SBSB and sleep habit. Regarding PA, 36.5% of the students who were not involved in PA were found to have depressive symptom, A statistically significant (χ^2^ = 25.84; *p* < 0.001) relationship was found between involvement in PA and depressive symptom. Besides, 36.3% of the respondents who were not involved in regular PA had depressive symptom, while 26.2% who were involved in regular PA were also found to have depressive symptom, and there is a significant (χ^2^ = 26.09; *p* < 0.001) relationship between this variable and depressive symptom. Again, 34% of the respondents who did PA <30 min per day were found to have depressive symptom; in contrast, 23.1% of the respondents who did PA 30–60 min per day as well as 24.2% of the respondents who did PA more than 60 min per day also reported to be suffering from depressive symptom. The relationship between duration of the daily PA and depressive symptom was found to be statistically significant (χ^2^ = 28.36; *p* < 0.001). Furthermore, 44.9% of the respondents who reported doing PA in the evening had depressive symptom. On the other hand, 26.2% doing PA early in the morning and 22.5% doing PA in the late afternoon had depressive symptom. PA time was found to have a statistically significant (χ^2^ = 57.50; *p* < 0.001) association with depressive symptom.

**Table 2:** Association of lifestyle variables with students' depressive symptom.

**Variables**	**Depressive symptom^a^**	
	**Frequency (% within variable)**	**χ^**2**^ value (*p*)**
**Physical activity (PA)**		
**Involved in PA**		
Yes	389 (26.4)	25.84 (<0.001)^**^
No	307 (36.5)	
**Regular PA**		
Yes	374 (26.2)	26.09 (<0.001)^**^
No	322 (36.3)	
**Duration of daily PA (*****n*** **=** **1,472)**		
<30 min/day	491 (34.0)	28.36 (<0.001)^**^
30–60 min/day	122 (23.1)	
>60 min/day	83 (24.2)	
**PA time (*****n*** **=** **1,472)**		
Early morning of the day	124 (26.2)	57.50 (<0.001)^**^
Late afternoon of the day	179 (22.5)	
Evening of the day	71 (44.9)	
**Screen based sedentary behavior (SBSB)**		
**Use of social media (e.g., Facebook)**		
Yes	474 (33.3)	17.76 (<0.001)^**^
No	222 (25.0)	
**Screen based recreation (Movie, video game etc.)**		
Yes	637 (30.0)	0.06 (0.801)
No	59 (30.9)	
**Duration of daily SBSB**		
≤2 h/day	322 (24.5)	45.88 (<0.001)^**^
>2 h/day	374 (37.5)	
**Sleep quality**		
**Satisfaction about daily sleep**		
Yes	315 (20.7)	189.43 (<0.001)^**^
No	381 (48.4)	
**Sleep habit**		
Short sleep duration (<7 h/day)	311 (34.2)	21.32 (<0.001)^**^
Ideal sleep duration (7–9 h/day)	330 (26.2)	
Long sleep duration (>9 h/day)	55 (38.5)	

a *The cut-off of PHQ-9 ≥10 is used for analysis*.

** *The p value is significant at p < 0.01*.

Regarding the SBSB, 33.3% of the respondents who stated using social media (e.g., Facebook) were found having depressive symptom, while 25.0% of them who were not using any type of social media also reported to be suffering from depressive symptom. Using social media had a significant statistical (χ^2^ = 17.76; *p* < 0.001) relationship with depressive symptom. Similarly, in this study, 37.5% of the respondents with a reported daily screen time of >2 h per day as well as 24.5% with a daily screen time of ≤2 h per day were found to have depressive symptom. The duration of daily SBSB had a significant (χ^2^ = 45.88; *p* < 0.001) statistical relationship with depressive symptom. In addition, 48.4% respondents who had dissatisfaction about their daily sleep reported having depressive symptom, while 20.7% respondents who were satisfied with their daily sleep also reported suffering from depressive symptom, and there was a statistically significant (χ^2^ = 189.43; *p* < 0.001) link between sleep satisfaction and depressive symptom. Besides, sleep duration was also found to be statistically significant (χ^2^ = 21.32; *p* < 0.001) with depressive symptom. Of the respondents who reported a long sleep duration (>9 h/day), 38.5% reported suffering from depressive symptom. Again, 34.2% of the respondents with a short sleep duration (<7 h/day) and 26.2% with an ideal sleep duration (7–9 h/day) also reported suffering from depressive symptom.

### Association Between Predictive Study Variables and Depressive Symptom

Table 3 demonstrates the association between depressive symptom and predictive study variables. In this table, both bivariate and multivariate analyses of the variables are presented. Adjusted estimate-1 was adjusted for all socio-demographic variables (age, gender, grade, residence) and adjusted estimate-2 was adjusted for level of PA, duration of daily screen time, satisfaction about daily sleep, sleep habit, and perceived weight category. After adjusting for socio-demographic and lifestyle variables, the odds ratios for variables changed slightly.

**Table 3:** Association between predictive study variables and depressive symptom among school adolescents in Dhaka, Bangladesh^a^.

**Variables**	**Unadjusted estimates**			**Adjusted estimates-1^b^**			**Adjusted estimates-2^c^**		
	**Odds ratio**	**95% CI**	***p***	**Odds ratio**	**95% CI**	***p***	**Odds ratio**	**95% CI**	***p***
**Socio-demographic variables**									
**Gender**									
Female	1.70	1.42–2.04	<0.001	1.90	1.57–2.29	<0.001	1.67	1.34–2.04	<0.001
Male	1.00			1.00			1.00		
**Student grade**									
Class 10	1.63	1.28–2.07	<0.001	1.26	0.91–1.75	0.162	1.44	1.16–1.80	0.001
Class 09	1.44	1.17–1.77	0.001	1.35	1.06–1.73	0.014	1.40	1.08–1.82	0.012
Class 08	1.00			1.00			1.00		
**Age**									
≥15 years	1.57	1.31–1.88	<0.001	1.39	1.09–1.78	0.008	1.35	1.11–1.64	0.003
<15 years	1.00			1.00			1.00		
**Residence**									
Urban	2.29	1.82–2.88	<0.001	2.45	1.94–3.10	<0.001	1.42	1.10–1.83	0.007
Semi-urban	1.93	1.52–2.44	<0.001	1.88	1.47–2.40	<0.001	1.39	1.08–1.79	0.011
Rural	1.00			1.00			1.00		
**Lifestyle variables**									
**Involved in PA**									
No	1.60	1.33–1.92	<0.001	1.52	1.25–1.84	<0.001	1.44	1.14–1.84	0.003
Yes	1.00			1.00			1.00		
**Regular PA**									
No	1.60	1.33–1.92	<0.001	1.53	1.26–1.85	<0.001	1.43	1.12–1.82	0.004
Yes	1.00			1.00			1.00		
**Level of PA**									
Inactive/Low PA	1.67	1.38–2.03	<0.001	1.44	1.17–1.77	0.001	1.60	1.30–1.96	<0.001
Moderate to vigorous PA	1.00			1.00			1.00		
**Use of social media (e.g., Facebook)**									
Yes	1.50	1.24–1.80	<0.001	1.50	1.23–1.83	<0.001	1.16	0.91–1.46	0.229
No	1.00			1.00			1.00		
**Duration of daily screen time**									
>2 h/day	1.85	1.55–2.22	<0.001	1.74	1.44–2.11	<0.001	1.68	1.39–2.03	<0.001
≤2 h/day	1.00			1.00			1.00		
**Satisfaction about daily sleep**									
No	3.60	2.98–4.33	<0.001	3.14	2.58–3.82	<0.001	3.23	2.64–3.96	<0.001
Yes	1.00			1.00			1.00		
**Sleep habit**									
Short sleep duration (<7 h/day)	1.47	1.22–1.77	<0.001	1.30	1.07–1.58	0.007	0.98	0.80–1.21	0.856
Long sleep duration (>9 h/day)	1.76	1.23–1.53	0.002	1.77	1.22–2.57	0.002	1.47	0.99–2.17	0.054
Ideal sleep duration (7–9 h/day)	1.00			1.00			1.00		
**Body image dissatisfaction^d^**									
Yes	2.64	2.18–3.18	<0.001	2.40	1.98–2.92	<0.001	NA		
No	1.00			1.00					
**Perceived weight category**									
Over-weight/obese	2.52	2.02–3.13	<0.001	2.19	1.75–2.75	<0.001	2.14	1.70–2.70	<0.001
Underweight	2.86	2.14–3.82	<0.001	2.78	2.05–3.74	<0.001	2.30	1.70–3.13	<0.001
Normal	1.00			1.00			1.00		

a *Estimates are based on binary logistic regression with depressive symptom, PHQ-9≥10 scores, as dependent variable*.

b *Adjusted for all presented socio-demographic variables (age, gender, grade, residence) in the table*.

c *Adjusted for level of PA, duration of daily screen time, satisfaction about daily sleep, sleep habit, and perceived weight category*.

d *Body image dissatisfaction was highly correlated with perceived weight category (r = −0.88) and removed from the multivariate model*.

*NA Not applicable (excluded from the multivariate model since their P value was >0.10)*.

In the unadjusted estimate, it was found that female students were 1.7 times (95% CI: 1.42–2.04) more likely to be suffering from depressive symptom than male. Besides, in adjusted estimates-1 and adjusted estimates-2, it was found that female students had 1.9 (95% CI: 1.57–2.29) times and 1.67 (95% CI: 1.34–2.04) times higher risks of suffering from depressive symptom than males, respectively. Students of class 10 had 1.63 times (95% CI: 1.28–2.07) higher risks of suffering from depressive symptom than class 8 students in the unadjusted estimates. In adjusted estimates 1 and 2, compared to class 8 students, class 10 students were, respectively, at 1.26 times (95% CI: 0.91–1.75) and 1.44 times (95% CI: 1.16–1.80) higher risks of suffering from depressive symptom. Statistically significant higher odds ratio (OR: 1.44; 95% CI: 1.17–1.77) for depressive symptom was found among students of class 9 than among class 8 students in the unadjusted estimate. In adjusted estimates 1 and 2, it was found that class 9 students were, respectively, 1.35 times (95% CI: 1.06–1.73) and 1.40 times (95% CI: 1.08–1.82) more likely to be suffering from depressive symptom than class 8 students. In reference to respondents aged <15 years, respondents aged ≥15 years had 1.39 times (95% CI: 1.09–1.78) more risks of suffering from depressive symptom in adjusted estimate-1 and 1.35 times (95% CI: 1.11–1.64) more risks in adjusted estimate-2. Besides, respondents of this age group were found to be 1.57 times (95% CI: 1.31–1.88) more likely to be suffering from depressive symptom than others in the unadjusted estimate. With regard to residence, the unadjusted estimate shows that urban students were 2.29 times (95% CI: 1.82–2.88) more likely to be suffering from depressive symptom than rural students, while semi-urban students were 1.93 times (95% CI: 1.52–2.44) more likely to be suffering from depressive symptom than their rural counterparts. In adjusted estimate-1, urban students had 2.45 times (95% CI: 1.94–3.10) greater risks of suffering from depressive symptom than rural students, while for semi-urban students the risk was 1.88 times (95% CI: 1.47–2.40) higher than those for rural ones. Urban students and semi-urban students were, respectively, 1.42 times (95% CI: 1.10–1.83) and 1.39 times (95% CI: 1.08–1.79) more likely to be suffering from depressive symptom than rural students, as indicated in adjusted estimate 2.

In case of lifestyle variables, the unadjusted estimate shows that students who were not involved in PA were 1.60 times (95% CI: 1.33–1.92) more likely to be suffering from depressive symptom than those who were involved in PA, and same is the case with the variable—irregularity in PA. In adjusted estimate-1, it was found that the risk of depressive symptom was 1.52 times (95% CI: 1.25–1.84) higher in those not involved in PA than those involved in PA, and 1.53 times (95% CI: 1.26–1.85) higher in those irregular in PA than those who reported doing PA regularly. Again, adjusted estimate-2 shows that the students not involved in PA had 1.44 times (95% CI: 1.14–1.84) greater risks of depressive symptom than those involved in PA, and students who were irregular in PA had 1.43 times (95% CI: 1.12–1.82) greater risks of depressive symptom than those regular in PA. Students who were inactive or involved in low PA were 1.67 times (95% CI: 1.38–2.03) more likely to be suffering from depressive symptom than those involved in moderate to vigorous PA in the unadjusted estimate. In adjusted estimates 1 and 2, the students with inactivity or low PA were 1.44 times (95% CI: 1.17–1.77) and 1.60 times (95% CI: 1.30–1.96) more likely to have depressive symptom than those with moderate to vigorous PA. In addition, the risk of depressive symptom was 1.50 times (95% CI: 1.24–1.80) higher in students using social media than those not using any social media. Again, in the adjusted estimates, those who used social media were, respectively, 1.50 times (95% CI: 1.23–1.83) and 1.16 times (95% CI: 0.91–1.46) more likely to be suffering from depressive symptom than those who reported no use of social media. In the unadjusted estimate, a significantly higher odds ratio (OR: 1.85; 95% CI: 1.55–2.22) was found for depressive symptom among students with a daily screen time of >2 h than those with a lower screen time. Adjusted estimate-1 shows that students with a screen time of >2 h were 1.74 times (95% CI: 1.44–2.11) more likely to suffer from depressive symptom than those with a lower screen time, while in adjusted estimate-2, they were 1.68 times (95% CI: 1.39–2.03) more likely to be suffering from depressive symptom than those with a lower screen time.

In the unadjusted estimates, students with dissatisfaction about sleep were 3.60 times (95% CI: 2.98–4.33) more likely to suffer from depressive symptom than those who were satisfied with their daily sleep. Adjusted estimates show that daily sleep dissatisfaction was, respectively, 3.14 times (95% CI: 2.58–3.82) and 3.23 times (95% CI: 2.64–3.96) more likely to cause depressive symptom among students, compared to those satisfied with daily sleep. In reference to ideal sleep duration (7–9 h/day), students who reported short (<7 h/day) and long (>9 h/day) sleep duration had 1.47 times (95% CI: 1.22–1.77) and 1.76 times (95% CI: 1.23–1.53) higher risks of depressive symptom, respectively, in the unadjusted estimate. In adjusted estimate-1, students with a short sleep duration had 1.30 times (95% CI: 1.07–1.58) and students with a long sleep duration had 1.77 times (95% CI: 1.22–2.57) greater risks of depressive symptom than those with an ideal sleep duration. Besides, adjusted estimate-2 shows that students who reported long sleep duration had 1.47 times (95% CI: 0.99–2.17) greater risks of depressive symptom than those who reported an ideal sleep duration.

The unadjusted estimate shows that the students who reported body image dissatisfaction were 2.64 times (95% CI: 2.18–3.18) more likely to suffer from depressive symptom than those satisfied with their body image. However, adjusted estimate-1 shows that body image dissatisfaction was responsible for 2.40 times (95% CI: 1.98–2.92) higher risks of developing depressive symptom in students. In adjusted estimate 2, it was found that body image dissatisfaction was highly correlated with perceived weight category, and for this it was removed from there. Furthermore, in the unadjusted estimate, overweight/obese and underweight students were 2.52 times (95% CI: 2.02–3.13) and 2.86 times (95% CI: 2.14–3.82) more likely to be suffering from depressive symptom, respectively, than the students with normal body weight. Besides, adjusted estimate-1 shows that underweight and overweight/obese students were 2.78 times (95% CI: 2.05–3.74) and 2.19 times (95% CI: 1.75–2.75) more likely to suffer from depressive symptom, respectively, than those with normal body weight, where as adjusted estimate-2 shows that overweight/obese and underweight students had 2.14 times (95% CI: 1.70–2.70) and 2.30 times (95% CI: 1.70–3.13) greater risks of depressive symptom, respectively, than those with normal body weight.

## Discussion

This study indicates that the prevalence of moderate to severe depressive symptom among urban, semi-urban and rural school adolescents of the Dhaka district in Bangladesh is 30.1%. This prevalence is lower than that (36.6%) reported in the pilot study conducted upon the same population in urban and semi-urban areas in 2018 (18). This difference in the prevalence of adolescent depressive symptom in this study might result from the inclusion of school adolescents from the rural areas where depressive symptom is generally less prevalent than in urban or semi-urban areas due to the impact of some factors. However, the prevalence of depressive symptom in this study is much higher than that (14%) reported in a study conducted in 2012 among 2440 adolescents aged 13–19 years in Bangladesh (20) as well as than the prevalence (25%) reported in another study conducted among 898 Bangladeshi adolescents (19). Again, compared to this study, a much higher prevalence was reported in a 2014 study conducted among 165 male adolescents aged 15–19 in Bangladesh (49%) (28), in a 2017 study conducted among 1412 adolescents in Bihar, India (49%) (29) and in a 2015 study conducted upon 374 adolescents in Haryana, India (52.9%) (30). In a study conducted in Pakistan in 2016 on 204 adolescent girls, the prevalence of depression was 72% (31), which is quite higher than the prevalence of depressive symptom among girls (60.8%) in this study. Again, the prevalence of depressive symptom among adolescents in our study is quite lower than the prevalence reported in an Iranian study on 670 female school adolescents aged 15–18 years in 2016 (72.6%) (32).

Different factors played a role in the high prevalence of adolescent depression in these studies. For example, in the Iranian study various academic factors (e.g., school type and field of study) along with residential setting were responsible for the high prevalence (32). In the previously mentioned Pakistani and Indian studies, the contributing factor behind adolescent depression was low grades in the previous academic year. Furthermore, these studies reported some additional factors responsible for depression among adolescents, such as being a member of an ethnic minority, age, gender and religion (29–31).

In this study, numerous socio-demographic as well as lifestyle variables are found to be responsible for the high prevalence of depressive symptom among school adolescents of Dhaka district, Bangladesh. The findings of this study demonstrated that 60.8% of the female participants were found to have depressive symptom, and as the regression analysis showed, female adolescents were 1.70 times more likely to be suffering from depressive symptom than their male counterparts. This finding is consistent with the findings of the pilot study where 42.9% female adolescents reported depression with the OR of 2.18 (18). Again, one study conducted on Thai adolescents showed higher prevalence (16.5%) of depression among female than male (14.7%) adolescents (33). Besides, the prevalence of depressive symptom in female adolescents in the current study is also consistent with the finding of another contemporary study where the prevalence of depression among female adolescents was 52.6% (32). Several factors are responsible for the high prevalence of depressive symptom among female adolescents. The social structure puts immense pressure directly or indirectly on female adolescents as she passes through different physiological and mental changes. The society places her in a situation where she can only think of herself as nothing but a commodity. Further, she has a pressure on herself of getting married as soon as possible once she reaches puberty. At the same age, while a male adolescent enjoys greater freedom in doing certain things, a female adolescent often experiences the fear of being teased and thus socially stigmatized while passing through a street. The community might not provide the same educational or other facilities for a female adolescent, while her male counterpart might often have greater freedom of pursuing his dream. These are some of the pressing and practical factors through which female adolescents of low- and middle-income countries (LMIC) like Bangladesh go through, and these factors might play an important role behind the high prevalence of depressive symptom among them. However, among different socio-demographic causes, increasing age is another significant cause (OR: 1.57) behind depressive symptom among adolescents in the current study, and this finding is in sync with the findings of other studies (34, 35). Various factors are responsible for depressive symptom among adolescents aged ≥15 years including increased academic pressure, more time spent on screen based sedentary behavior (SBSB), family pressure, and so on. Students of higher grades or classes are found to have more depressive symptom in the present study: students of the 10th grade (OR: 1.63) and 9th grade (OR: 1.44) were found to suffer more from depressive symptom than the 8th graders. The reason behind this could be that in higher classes, academic pressure like pressure of positive academic attainment increases. During this time adolescents lack proper support and warmth from parents, and they suffer from depressive symptom (36). Again, there is evidence that the process of getting prepared for board final exam causes depressive symptom in adolescents (35). As the findings of this study suggest, urban adolescents were the largest in number (43.7%) and had the odds ratio 2.29 in case of suffering from depressive symptom, which is the highest in all residential settings. This finding is consistent with the finding of the pilot study, where 47.5% of the urban students (OR: 2.34) reported having depressive symptom, compared to students from the semi-urban areas (18). Besides, this finding is also in line with the findings of other studies (37, 38). Of the lifestyle-related factors associated with adolescent depressive symptom in this study, the most prominent ones were physical inactivity and short duration of PA. Findings from the regression analysis show that these factors had a significant association with depressive symptom among adolescents. Studies have demonstrated that there is a negative relationship between PA and depression (39, 40).

The current study revealed that 33.3% of students who reported to use social media (e.g., Facebook, Instagram or Twitter) were found to be suffering from depressive symptom, and the regression analysis showed that social media use was significantly (OR: 1.50) associated with depressive symptom. This social media use leads to increase in daily SBSB of adolescents. This study found that adolescents with SBSB of >2 h per day had 1.85 times higher risks of suffering from depressive symptom. Social media use also affects the sleep habit of adolescents. In this study, those who were dissatisfied with their daily sleep were 3.60 times more likely to suffer from depressive symptom than those satisfied with their daily sleep. Vidal et al. (41) in their study stated that increased social media use led to a decrease in face-to-face interactions, an increase in addictive behaviors as well as an increase in online bullying, social pressure and social comparison (41). When adolescents spend more time on social media, this hampers the quality of their sleep (42). Again, body image dissatisfaction, like perceiving self-body image as being obese or underweight, causes depressive symptom among adolescents. The current study reported that body image dissatisfaction like perceiving oneself as overweight or underweight significantly affected depressive symptom among Bangladeshi adolescents. Due to pubertal development during adolescence, adolescents, particularly females, become dissatisfied with their body image. This body image dissatisfaction is greatly influenced by exposure to social media and social acceptance from peers. During adolescence, body image dissatisfaction compels the adolescents to be socially isolated due to the absurd presentation of ideal body image (43).

### Strength and Limitations

Notwithstanding the gravity of this public health issue, very few studies have been conducted upon adolescent mental health in Bangladesh. These studies were conducted either in urban or rural settings, not in urban, semi-urban and rural area simultaneously. To the best of our knowledge, this is the first study to explore depressive symptom among urban, semi-urban and rural adolescents of Bangladesh. Spanning over a period of 1 year, this study followed a pilot study conducted upon urban and semi-urban adolescents of the study area (18). Therefore, the findings of this study could be utilized in making policy decisions to address adolescent mental health diseases in the country. Again, this study maintained a very comprehensive and rigorous field implementation.

This study has its limitations as well. This cross sectional study collected data at one point of time and the participants represent a small segment of the adolescents of the Dhaka district, which is why the findings of this study cannot be generalized for other districts of the country and instead should be used with caution. Besides, the participants' self-reported information involves the risk of recall bias. Therefore, large-scale longitudinal studies need to be conducted on the same cohort covering the entire Dhaka district to advance generalizable findings that would feed into policy formulation initiatives in Bangladesh.

## Conclusions

The findings of this study revealed that a substantial number of Bangladeshi adolescents are experiencing depressive symptom and different socio-demographic (e.g., being female, living in urban area etc.) and lifestyle-related factors (e.g., not doing PA, sleep dissatisfaction etc.) play active roles as contributing factors behind this phenomenon. With a severe dearth in evidence, these findings are crucial to stress the importance of conducting longitudinal research to explore issues related to adolescents' mental health and well-being in the country. These findings might help to design low intensity psychosocial interventions for this particular subgroup of the population. Moreover, these findings might be utilized in formulating appropriate adolescent friendly policies for the betterment of the mental health condition of the future generations of Bangladesh.

## Data Availability Statement

The original contributions presented in the study are included in the article/supplementary material, further inquiries can be directed to the corresponding authors.

## Ethics Statement

The studies involving human participants were reviewed and approved by Institutional Review Board, Jahangirnagar University [Ref No: BBEC, JU/ M/ 2019(8)3]. Besides, all participants read, understood and signed a written consent form at the time of survey data collection. Written informed consent to participate in this study was provided by the participants' legal guardian/next of kin.

## Author Contributions

AA: conceptualization, methodology, investigation, data curation, formal analysis, writing—original draft, writing—review and editing, and validation. SH: conceptualization, investigation, data curation, writing—review and editing, and validation. MH and MU: writing—review and editing and validation. SA: writing—original draft and validation. MS: conceptualization, supervision, writing—review and editing, and validation. All authors contributed to the article and approved the submitted version.

## Funding

This study was funded by Ministry of Science and Technology of the People's Republic of Bangladesh (NST 2019-20/MSc).

## Conflict of Interest

MH was employed by company Jeeon Bangladesh Ltd. The remaining authors declare that the research was conducted in the absence of any commercial or financial relationships that could be construed as a potential conflict of interest.

## Publisher's Note

All claims expressed in this article are solely those of the authors and do not necessarily represent those of their affiliated organizations, or those of the publisher, the editors and the reviewers. Any product that may be evaluated in this article, or claim that may be made by its manufacturer, is not guaranteed or endorsed by the publisher.
